# Traumatic bilateral L4-5 facet fracture dislocation: a case presentation with mechanism of injury

**DOI:** 10.1186/s12891-019-2921-5

**Published:** 2019-11-23

**Authors:** Kevin Chi Him Fok, Jason Pui Yin Cheung

**Affiliations:** Department of Orthopaedics and Traumatology, The University of Hong Kong, Pokfulam, Hong Kong SAR, China

**Keywords:** Traumatic spondylolisthesis, Locked facet, Jumped facet, L4–5

## Abstract

**Background:**

Traumatic bilateral locked facet joints at L4–5 level are a rare entity. A careful review only revealed four case reports. This case presented with an unusual mechanism of injury.

**Case presentation:**

We present a case of a 40-year-old male who suffered bilateral L4–5 traumatic facet fracture dislocation following a fall injury. The dislocation was associated with fractures of bilateral L4 inferior articular processes, left L4 pedicle, L4 spinous process and postero-inferior body of L4. He presented with cauda-equina syndrome and underwent emergency decompression, reduction and instrumented fusion.

**Conclusion:**

The biomechanics of the lumbar spine may differ with each individual. L4–5 dislocation may be a variant to lumbosacral (L5-S1) dislocation, owing to hyperextension injury.

## Background

Traumatic facet dislocation in the lower lumbar spine is rare, with only handful of reported cases with lumbosacral (L5-S1) dislocation. We present a case of a young male with bilateral L4–5 traumatic facet dislocation after a fall injury.

## Case presentation

A 40-year-old male was involved in an industrial accident. He fell backwards with his back landing onto a metal bar, while another person landed on his thighs which caused a hyperextension moment across his lumbar spine. He experienced immediate pain over the lower back with numbness in bilateral legs.

**Fig. 1:**
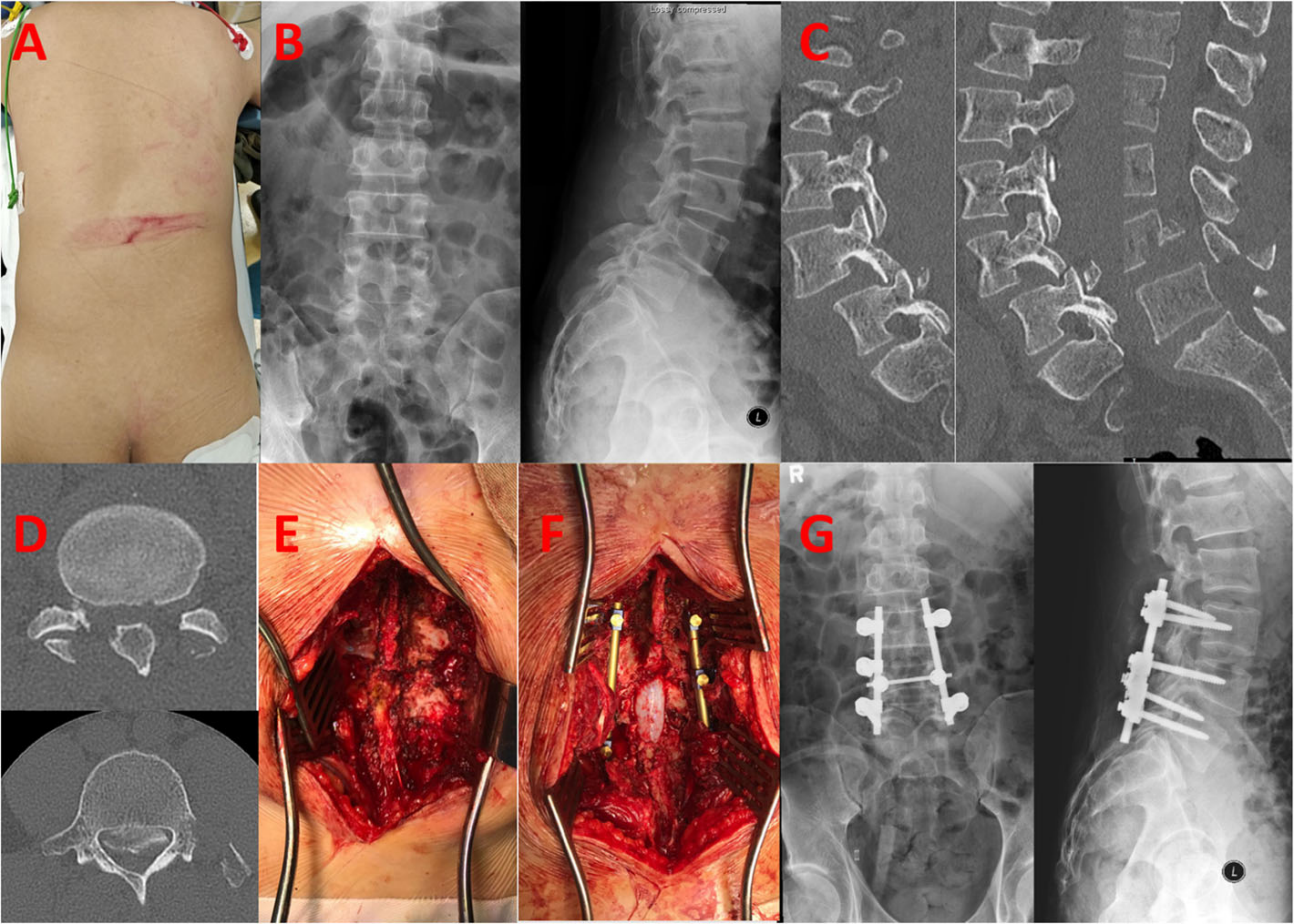
**a** Collision site of metal bar as fulcrum. **b** XR showing grade II spondylolisthesis of L4 on L5, fracture dislocation of facet joints, and fractures of left transverse processes from L1 through L4, a fracture of posterior inferior L4 vertebral body, spina bifida occulta of L5. **c**, **d**. CT scan showing fracture of bilateral L4 inferior articular processes, fracture of left L4 transverese process, left L4 pedicle and the L4 spinous process, L4 vertebral body fragment retropulsion causing severe spinal canal narrowing, and coronally oriented L4/5 facets. **e** Spontaneous reduction of dislocation upon prone position. **f** Laminectomy and posterior spinal fusion with instrumentation. **g** Post-operative XR showing reduction of dislocation

Physical examination upon arrival showed weakness of both lower extremities. The hip flexion (L2) and knee extension (L3) were Medical Research Council (MRC) grade 2/5 on both sides, and ankle dorsiflexors (L4), long toe extensors (L5) and ankle plantar flexors (S1) were grade 0/5 on both sides. There was absent of sensation by light touch and pin prick over L5 to S1 dermatomes on both sides. The lower limb reflexes were absent. Per-rectal examination showed absence of deep anal pressure, peri-anal sensation and voluntary grip. Clinically, the patient had cauda equina syndrome. There was a horizontal patch of bruising over the thoracolumbar region of the back, resulting from the collision with a metal bar (Fig. 1a).

X-ray and Computed tomography showed grade II traumatic spondylolisthesis of L4 on L5, fracture dislocation of facet joints, and fractures of left transverse processes from L1 through L4, left L4 pedicle, L4 spinous process and the posterior inferior L4 vertebral body (Fig. 1b). There was retropulsion of a L4 vertebral body fragment, causing > 50% spinal canal narrowing. (Fig. 1d).

Emergency surgery was performed within 6 h of injury. There was spontaneous reduction of the L4-5 dislocation upon prone position (Fig. 1e), the supraspinous ligament was not disrupted from L3 to S1, and the facet capsules of L2–3 and L5-S1 were preserved. Pedicle screws were inserted to bilateral L3, right L4 and bilateral L5. Laminectomy was performed from L3 to L5 for decompression (Fig. 1f). Posterolateral fusion was performed with autogenous bone graft and tricalcium phosphate. (Fig. 1g).

Post-operative magnetic resonance imaging did not show any disruption of disc or anterior longitudinal ligaments, and the central canal was well decompressed. At 9 months post-injury, he had regained sphincter control and was able to walk with frame with full neurological recovery over the right side. However, his distal muscle groups over the left side remained weak with grade 2/5 ankle dorsiflexors, long toe extensors and ankle plantarflexors.

## Discussion

Bilateral facet joint dislocation of L4–5 level is a rare entity, a PubMed search revealed only four reports [1–4] (Table 1), while most were based on lumbosacral (L5-S1) dislocation since described by Watson-Jones in 1940 [5, 6]. Although Watson-Jones described the hyperextension stress in the first case of reported lumbosacral dislocation, most authors have considered the main mechanism to be hyperflexion [5]. It is not until recently, that the pathophysiology of traumatic dislocations of the lumbar spine are again considered to be due to hyperextension injury [2, 4, 5].

**Table 1:** Previous reports of L4-5 facet joint dislocation

Author (year)	Patient (age/sex)	Description of injury	Presenting symptoms	Bony injury	Soft tissue injury	Operation	Outcome	Proposed mechanism
Mori [1] (2002)	32/F	MVA, head-on collision Seatbelt worn	Lower back and abdominal pain. No neurological deficit	Bilateral L4–5 facet dislocation, Fractured right L4 and L5 transverse processes	Posterior longitudinal ligament disrupted. capsules torn, supra/interspinous ligaments torn, ligamentum flavum partially ruptured Dura intact	Reduction by resecting upper facet of L5, PSF	1.5 years: no low back pain, no neurological deficit	Hyperflexion injury, fulcrum at L4–5 level Weak lumbar paravertebral muscles to prevent sudden violent flexion and distraction
Song [3] (2005)	47/F	MVA, head-on collision. Seatbelt worn	Low back pain, No neurological deficit	Bilateral L4–5 facet dislocation, Fractured left L5 transverse process, Anterosuperior fracture fragment of L5 body	Ruptured posterior ligament complex and posterior muscles Disrupted capsules. Dura intact	Open reduction, Laminectomy, Posterior interbody fusion with iliac bone PSF, PLF	10 months: Fusion achieved	L4–5 facet located more sagittally, L5 more stable due to binding of iliolumbar and sacroiliac ligaments
Deniz [2] (2008)	44/M	MVA, crashed into a tree, thrown onto the ground	Low back pain, Numbness and weakness in both extremities, claudication symptoms	Bilateral L4–5 facet dislocation, Fractured bilateral L4 inferior articular processes	L4–5 foraminal disc herniation	Open reduction, Decompression, Posterior interbody fusion with cages PLF, PSF	3 months: symptoms free	Extension and axial load
Zenonos [4] (2016)	36/M	MVA, head-on collision. Seatbelt worn	No neurological deficit	Bilateral L4–5 facet dislocation, Fractured right L5 superior articular process, Fractured right L1-L5 transverse processes Fractured L4 spinous process Anterosuperior fracture fragment of L5 body	Ruptured L3-S1 interspinous and supraspinous ligaments, Ruptured posterior and anterior longitudinal ligaments, L4–5 disc rupture	Reduction by removing superior L5 facets, Laminectomy, PSF, PLF	3 months; no back pain, no neurological deficit	Extension-distraction forces

*MVA* motor vehicle accident, *PSF* posterior spinal fusion, *PLF* posterolateral fusion

The mechanisms of injury in the reported cases of L4–5 dislocation were high energy motor vehicle accidents with head-on collisions, where three out of four had usage of seatbelt, and one thrown out of the vehicle. These were associated with significant posterior soft tissue injuries, including supraspinous ligaments, interspinous ligaments and facet capsules of other levels [1–4] (Table 1). Zenonos [4] proposed the pathophysiology of the injury as such: a seatbelt holds down the thoracic spine and pelvis by the shoulder harness and waist harness respectively. With the thoracic spine as a fulcrum, the forward momentum of the body and remaining thoracolumbar spine forces the spine to swing forward. The greater distance from the fulcrum, the greater the force due to a longer moment arm. At the same time, the pelvis in splinted down by waist harness and immobilized, hence there is a large extension-distraction force at the lower lumbar and lumbosacral junction. However, high energy injuries are always complex and may be difficult to analyse and to deduce a single injury pattern.

**Fig. 2:**
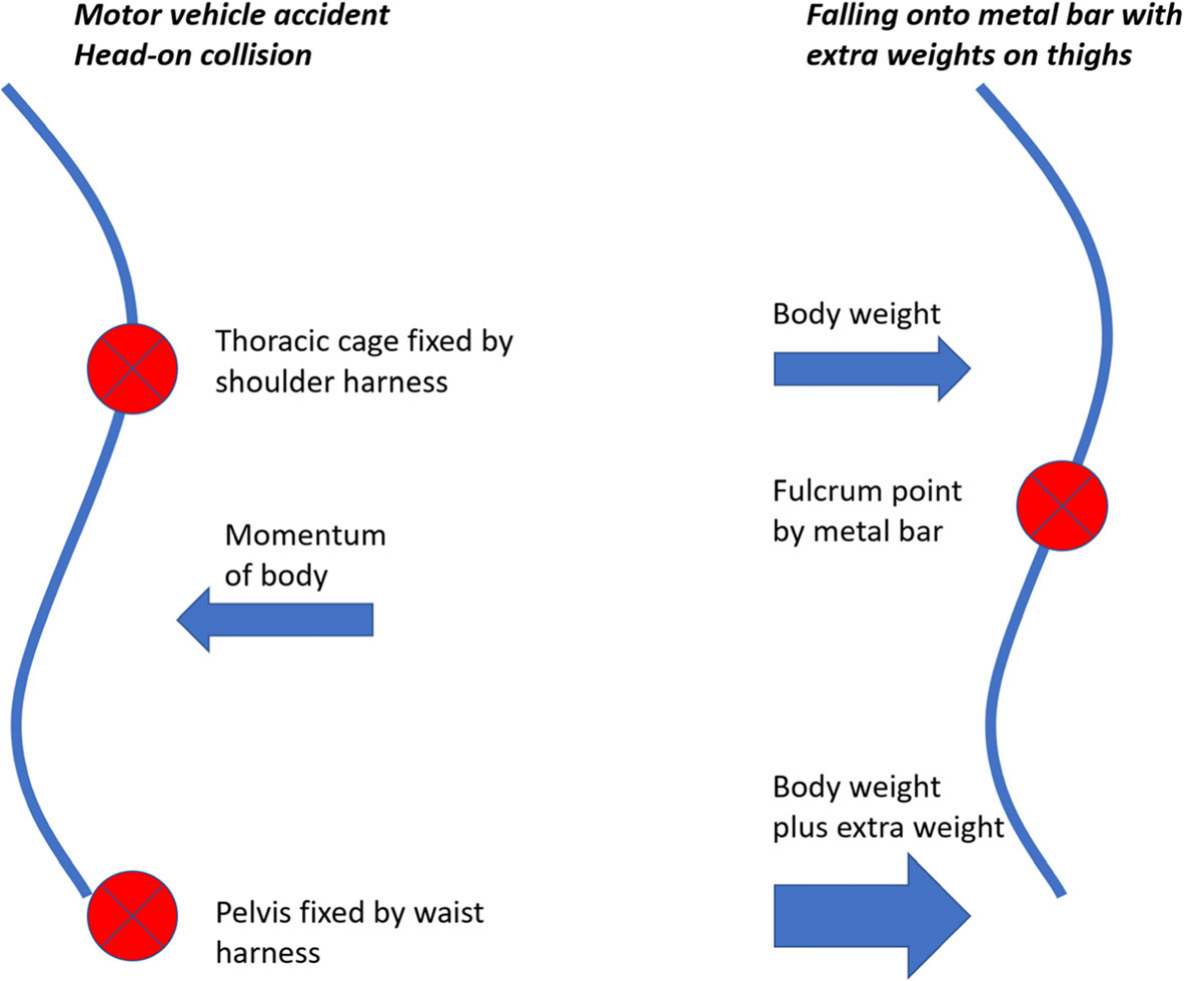
Our patient had a similar mechanism of injury as a motor vehicle accident with head-on collision. The points of fixation by seatbelts are replaced by body weights and the momentum of the body is replaced by a fulcrum at the metal bar. Both situations result in a forward motion of the lumbar spine relative to the remainder of the body with hyperextension of the lumbar spine

Our case had a similar mechanism of injury but resulting from a low energy falling accident. This provides more insight on the pathophysiology of such injuries. Our patient fell with his back landing onto a metal bar with additional weights on his thighs. In this scenario, the metal bar acted as a fulcrum, with the spinal segments above and below translating posteriorly after the impact. The additional weight on his thigh forced the lumbar spine into hyperextension, which further propagated the injury. When compared to the motor vehicle accidents described previously, the relative vectors are the same. The points of fixation by seatbelts are replaced by body weights, while the momentum of body replaced by the fulcrum represented by the metal bar; both situations results in a forward motion of the lumbar spine relative to the remainder of the body, resulting in hyperextension of the lumbar spine (Fig. 2).

In the current case, the injury pattern suggested that the traumatic force is exerted by extension stress. There are fractures of the L4 spinous process, bilateral L4 inferior articular processes and L4 postero-inferior body. This is further supported by fractured transverse processes from L1-L4 as an indirect evidence of strong contractions of iliopsoas muscles to resist hyperextension. Also, the supraspinous ligament was not disrupted from L3 to S1, and the facet capsules of L2–3 and L5-S1 were preserved, hence a flexion injury is less likely. During a hyperextension injury, posterior structures experience greater compressive forces. Therefore, the spinous process fractures first, then the facet joints, then the posterior body. The soft tissue damage is secondary to the dislocation, including the disruption of joint capsules and ligamentum flavum. The extensive posterior soft tissue damage in the reported cases may be due to a hyperflexion following the hyperextension, similar to a whiplash injury of the cervical spine but “reversed” due to the points of immobilization by the seatbelts as mentioned above.

Regarding the possible level of injury, Zenosos [4] proposed that the L4–5 is biomechanically more susceptible to dislocation when compared to L5-S1 for the following reasons: 1. The extension range of motion of L4–5 is less than L5-S1. 2. The L4–5 facet joints are oriented more sagittally when compared to L5-S1 facet joints. 3. The L5-S1 articular complex has a stronger ligamentous support.

However, this seems contrary to the majority of cases which the dislocation occured at the L5-S1 level. There are no biomechanical studies to confirm the exact ranges of flexion or extension of individual levels. It has been shown that the facet angle consistently increased from L2-L3 to L5-S1 with regards to the sagittal plane [7]. However, it has also been demonstrated that the L4–5 and L5-S1 facet joints have high variations in facet angle depending on ethnicity [8–11].

Our patient had spina bifida occulta at L5 (Fig. 1b), which may result in a higher extension range in L5-S1 than L4–5. Hence, a larger extension reserve for hyperextension, as there is no spinous process at the posterior of L5 to restrict extension. Also, his L4-L5 facet joints are almost in a coronal plane similar to L5-S1 facet joint (Fig. 1d). The above reasons may account for why the dislocation occurred at the L4–5 level rather than at L5-S1 resulting in a complex fracture dislocation.

We believe that the L4–5 traumatic facet fracture dislocation and the lumbosacral dislocation should be grouped into the same disease entity, as the mechanism of injury is similar. The level of injury differs in each individual as the anatomy of posterior structures is different. We have presented a case that has an anatomical variant which may have possibly explained why his injury has led to a injury at the level of L4-L5 rather than the lumbosacral junction. A spine is only as strong as its weakest link.

## Data Availability

Data sharing is not applicable to this article as no datasets were generated or analysed furing current study.
